# Insomnia and distress as mediators on the relationship from cyber-victimization to self-reported psychotic experiences: a binational study from Tunisia and Lebanon

**DOI:** 10.1186/s12888-023-05019-w

**Published:** 2023-07-20

**Authors:** Feten Fekih-Romdhane, Manel Stambouli, Diana Malaeb, Nour Farah, Majda Cheour, Sahar Obeid, Souheil Hallit

**Affiliations:** 1 The Tunisian Center of Early Intervention in Psychosis, Department of Psychiatry “Ibn Omrane”, Razi hospital, Manouba, 2010 Tunisia; 2 Faculty of Medicine of Tunis, Tunis El Manar University, Tunis, Tunisia; 3 College of Pharmacy, Gulf Medical University, Ajman, United Arab Emirates; 4 School of Pharmacy, Lebanese International University, Beirut, Lebanon; 5 Faculty of Science, Lebanese University, Fanar, Lebanon; 6 School of Arts and Sciences, Social and Education Sciences Department, Lebanese American University, Jbeil, Lebanon; 7 School of Medicine and Medical Sciences, Holy Spirit University of Kaslik, P.O. Box 446, Jounieh, Lebanon; 8 Research Department, Psychiatric Hospital of the Cross, Jal Eddib, Lebanon; 9 Applied Science Research Center, Applied Science Private University, Amman, Jordan

**Keywords:** Psychotic experiences, Insomnia, Cyber-victimization, Psychological distress, Psychosis risk

## Abstract

**Background:**

While expansive research has accumulated concerning the association between traditional, face-to-face peer victimization and psychosis, a paucity of empirical research has been undertaken so far to investigate these associations with experiences of new and evolving ways of victimization through the digital world. Exploring these associations is highly relevant and timely, given that emerging adults are heavy users of digital technologies, highly exposed to online risks, and are at the peak age of onset of psychosis. This study aimed to test the hypothesis that psychological distress and insomnia symptoms have a significant indirect mediating effect on the association between cyber-victimization and self-reported positive psychotic experiences (SRPEs) in a binational sample of Tunisian and Lebanese community adults.

**Method:**

The total sample was composed of 3766 participants; 3103 were from Lebanon (Mean age: 21.73 ± 3.80 years, 63.6% females) and 663 from Tunisia (Mean age: 26.32 ± 4.86 years, 59.9% females). Online anonymous self-report questionnaires were administered to all participants.

**Results:**

Higher SRPEs were found in Lebanese participants compared to Tunisians, in single participants compared to married ones, in those with a university level of education compared to secondary or less, in those who live in rural areas compared to urban, in those who do not smoke, do not drink alcohol and do not use marijuana or any other illegal drug. Furthermore, more cyber-victimization, a higher insomnia severity and psychological distress were significantly associated with higher SRPEs. After adjusting for potential confounders, mediation analysis demonstrated that higher cyber-victimization was significantly associated with more insomnia severity/psychological distress; which were, in turn, significantly associated with greater SRPEs. Finally, more cyber-victimization was significantly and directly associated with more positive dimension.

**Conclusion:**

Identifying insomnia and distress as mediators could provide novel insight for psychosis prevention efforts and intervention targets for cyber-victimized individuals prone to experience subclinical psychotic symptoms.

## Background

The dimensional approach states that the psychosis phenotype exists on a continuum across the community, with psychotic experiences (PEs) being at the milder end, and severe debilitating psychotic disorders being at the highest end of the continuum [1, 2]. PEs refer to subclinical psychotic symptoms (perceptual abnormalities and delusional beliefs) seen in non-clinical individuals, that could cause distress and interfere with daily functioning, but generally do not motivate help-seeking [3–5]. There is sufficiently strong epidemiological evidence that PEs are relatively highly prevalent in general population samples [6–9]; and are implicated in predicting other psychopathology and behavioral problems’ onset [10, 11], thereby contributing to high mental health services use and increased healthcare costs [12, 13].

Different methodologies exist for evaluating PEs. PEs can either be assessed by self-report (reflecting clinically non-confirmed self-reported PEs) and/or structured clinical interviews (reflecting clinically relevant PEs) [6]. Both methods have been shown to describe phenotypes pertaining to the same spectrum phenotype (i.e., clinical psychosis) [14]. Indeed, several validation studies provided evidence for the predictive validity of self-report measures of PEs against clinical judgment (e.g., [15, 16]). We focused in the present study on self-reported PEs (SRPEs). More recent research demonstrated that individuals who self-reported frequent and distressing SRPEs, even when not validated in structured clinical assessments, were associated with increased risk of mental health problems; which suggests that both SRPEs and PEs assessed by clinical judgment reflect the same underlying construct [14]. The SRPEs construct was found to be associated with family history for psychotic disorders and environmental risk factors for psychosis [17, 18]. In addition, baseline SRPEs have consistently been shown to confer an increased risk for developing a psychotic disorder outcome [6]; thus representing an important clinical phenotype for early intervention [14]. According to the psychosis proneness-persistence-impairment model by van Os et al. [3], PEs represent a “transitory developmental expression of psychosis” that might change over the individual life span to become abnormally persistent and impairing, depending on additional environmental exposure interacting with genetic risk. Interestingly however, a recent Swedish twin study [19] demonstrated that exposure to negative environmental factors (such as bullying) plays a greater role in the etiology of PEs than genetic factors, which further supports the major importance of the diathesis-stress model [20] for understanding PEs. Identifying how environmental factors can affect SRPEs can advance our knowledge of the mechanisms underlying psychosis proneness, and provide novel perspectives for prevention and early intervention strategies in psychosis. To this end, this study proposes to investigate the interplay between cyber-victimization and SRPEs.

### The relationship between cyber-victimization and SRPEs

Cyber-victimization (also called Internet or electronic victimization) is a new form of peer victimization referring to repeated and willful harassment (e.g., nasty comments, threats, humiliation, or exclusion) inflicted through information and communication technologies [21–24]. Cyber-victimization is a highly prevalent problem worldwide, affecting up to 73.5% adolescents and young adults [25, 26]. A growing body of knowledge suggests that cyber-victimization tend to target same victims as traditional bullying [27]; and that reported detrimental impact of both forms of victimization appears similar, such as suicidality [28], depression, low self-esteem [29], feeling unsafe at school, conduct problems, hyperactivity and peer problems [30], psychosomatic problems [30], and substance use [31]. This has led some authors to suggest that cyber-victimization can be viewed as “an extension” of face-to-face bullying victimization [32]. However, approaching the concept of cyber-victimization as a sub-category of in-real-life victimization might result in drastically underestimating its prevalence and negative consequences on mental health [33]. For example, while extensive evidence has shown that traditional bullying victimization increases the risk of later development of psychotic symptoms [34–37], such evidence is lacking for cyber-victimization. There is some emerging evidence in favor of differential effects of cyber- and traditional victimization [38]. There appears to be a worse impact on mental health and greater threats to psychosocial adaptation caused by cyber forms of victimization compared to traditional ones [39–41]. These data highlight the need for investigating the traditionally well-established relationship bullying-psychosis [34, 36, 42–44] with the new, online form of victimization.

Scant research has focused on the relationship between cyber-victimization and the psychotic phenomena in clinical and non-clinical populations. We could identify only four studies focusing on the relationship between cyber-victimization and psychotic symptoms [45–48]. Two of these studies specifically focused on SRPEs. The first study found that being a cyberbully or cyber-victim was associated with more severe SRPEs in healthy adolescents [47]. Similarly, the second study showed that being involved in cyberbullying was associated with greater psychoticism [48]. Multiple potential theoretically-driven mechanisms could be advanced to explain the relationship between cybervictimization and psychosis. The first theoretical explanation stipulates that some personal characteristics (e.g., pre-existing adjustment difficulties [49, 50]; impaired socio-emotional skills [51, 52]; attachment adversity [53, 54]) might enhance the likelihood of both being victimized and developing psychosis. The second hypothesized mechanism is that cyber-victimization and psychosis share a number of environmental factors, such as a precarious socioeconomic status [55, 56], low social support [57, 58]. Another mechanism can be suggested by analogy based on twin studies that documented shared genetic factors influencing risks for experiencing peer victimization and developing later SRPEs (e.g., [59]); but such evidence has yet to be demonstrated for cyber forms of victimization. Finally, plausible biological mechanisms to explain pathways linking cybervictimization to psychosis can be proposed, such as a disrupted functioning of the hypothalamic-pituitary-adrenal (HPA) axis [60, 61]. Overall, very little is known about the nature and mechanisms behind the relationship cybervictimization-SRPEs. To gain insight into the possible underlying pathways of this relationship, we hypothesized that psychological distress (i.e., depression, anxiety, stress) and insomnia symptoms have an indirect effect on the association between cyber-victimization and positive SRPEs.

### Distress and insomnia as mediators

Cyber-victimization seems to be a marker of more severe psychological distress. For instance, a recent metaanalysis encompassing 42 studies and 266,888 individuals (aged 8–20 years) estimated that cyber-victimization is associated with a unique 3.38-fold increased risk of depression [62]. Other meta-analyses’ findings have indicated that cyberbullying leads to anxiety symptoms [63]. It has also been stated that cyber-victimization poses a threat to belonging [64], contributing, in turn, to increased levels of stress [65]. A study found, for example, that being cyber-victimized (i.e., verbally harassed and socially excluded) has been associated with acute stress reactions [66]. Additionally, a longitudinal study demonstrated that cyber-victimization predicted depression and anxiety at 12 months follow-up, and that the positive prospective link between cyber-victimization and subsequent depression was stronger in individuals who experienced high perceived stress [66]. At the same time, psychological distress was shown to be significantly and positively correlated with increased psychosis risk, subthreshold psychotic symptoms, and an elevated risk of transition from high-risk states to a sustained psychosis [67, 68]. In particular, there is evidence from longitudinal research suggesting that depression is associated with subsequent psychotic symptoms in both clinical [69] and non-clinical populations [70, 71].

On the other hand, it has been argued that individuals targeted by different sorts of intended online aggressions, especially in the hours before bed, may struggle with initiating or maintaining sleep [72]. A large population-based study among Finnish adolescents found that being a cyber-victim was significantly associated with sleep problems [73]. A French study found that cyber-victimized high school adolescents reported significantly higher levels of insomnia than controls [74]. A Canadian longitudinal study found that adolescents who newly experienced cyber-victimization became more likely to report insufficient sleep duration at follow-up [75]. Similarly, a large national study among US high school students showed that electronically bullied females had inadequate sleep duration (fewer than 8 h on an average school night) [76]. Furthermore, there is consistent evidence that insomnia and sleep deprivation might precede, maintain or worsen psychotic symptoms over time [77–79]; and even precipitate the onset of psychotic episodes [80]. Some observations have also been made on the emergence of de novo perception abnormalities (e.g., distortions and hallucinations) in sleep deprived individuals with no history of psychiatric illness [81]. Insomnia has, for example, been associated with a 2- to 4-fold increase in the frequency of hallucinations in healthy individuals from the general population [82]. A multi-country study revealed that sleep disturbances were significantly linked to increased odds for at least one psychotic symptom [83].

### The present research

The purpose of this paper is to contribute to the existing literature in more than one way. First, while expansive research has accumulated concerning the association between traditional, face-to-face peer victimization and psychosis among healthy emerging adults [34, 36, 42–44], a paucity of empirical research has been undertaken so far to investigate these associations with experiences of new and evolving ways of victimization through the digital world (e.g., [45–48]). Exploring these associations is highly relevant and timely, given that emerging adults are heavy users of digital devices and technologies [84–86], highly exposed to online risks and cyber forms of victimization [25, 26], and are at the peak age of onset of psychotic disorders [87]. Second, this study intends to provide the scientific community with a better understanding of the factors underlying the relationship between cyber-victimization and SRPEs. Determining the influence of mediators may help elucidate the relationship between these constructs, and assist in designing and implementing evidence-informed prevention and intervention strategies. Third, most of the available studies on the association victimization-psychosis (and more particularly, cybervictimization-psychosis) have emerged from Western countries, with no studies identified from the Middle Eastern and North African (MENA) countries. The prevalence, experiences, consequences and reactions to being cyber-victimized vary widely across cultures [88–90]. Similarly, the prevalence and features of SRPEs are culturally-dependent [91, 92], and seem to be over-represented in Arab populations [93, 94]. Hence the importance of international studies on the topic from two lower-middle income developing Arab countries of the MENA region, Tunisia (North African) and Lebanon (Middle Eastern). In this regard, we performed the current study to test the hypothesis that psychological distress and insomnia symptoms have a significant indirect mediating effect on the association between cyber- victimization and SRPEs in a binational sample of Tunisian and Lebanese community adults.

## Method

The present study is part of a large cross-cultural, binational project including community-dwelling adults from Tunisia and Lebanon (The PEARLS project, [Psychotic Experiences in ARabs from Lebanon and tuniSia]). This project is aiming at validating the Arabic version of the Community Assessment of Psychic Experience (CAPE-42), as well as examining the nature and correlates of subclinical psychotic phenomena in these countries (for further details about the project, please see [95]). Participants have been invited to be part of our cross-sectional online study during June-September, 2022. Eligibility criteria involved: (1) being aged 18–35 years, (2) having no self-reported past personal history of physician-diagnosed mental illness, including psychosis, (3) having no prior antipsychotic drugs intake, and (4) willingness to participate. As such, participants with known mental disorders were excluded from our study. Data were collected using an online anonymous survey shared through social media networks (Facebook, Instagram, and WhatsApp). Detailed information about the purpose of the study were included in the informed consent form that was attached in the first page of the online survey. Participation was volunteer and no compensation was offered. We examined Internet protocol (IP) addresses in order to ensure that no respondent took the survey more than once. Ethics approval for this study was obtained from the Psychiatric Hospital of the Cross ethics committee (approval code: HPC-013-2022).

### Questionnaire

The questionnaire was presented in the native language (Arabic) of respondents, required 15–20 min to complete, and was divided into two sections. The first section comprised items about demographic information, including age, gender, marital status, educational level, housing area, living arrangement, and substance use. Participants were also asked whether they have been previously diagnosed by a physician with any mental illness, including psychosis, and if they had any prior antipsychotic drugs intake. The second section contained four self-report scales (The Revised Cyber Bullying Inventory–II [RCBI-II], the Community Assessment of Psychic Experience-42 [CAPE-42] scale, the Depression Anxiety Stress Scale 8 (DASS-8), and the Insomnia Severity Index [ISI]).

#### The RCBI-II

This is a 20-item, four-point Likert self-report measure involving two subscales to precise either the indicated cyberbullying behavior happened to the respondent as a cyber-victim (cyber-victimization subscale, 10 items), or was perpetrated by them as a cyberbully (cyberbullying subscale, 10-items) [96]. Only the cyber-victimization subscale was used in the context of the present study, with higher scores indicating exposure to greater cyber-victimization experiences. The Arabic version of the RCBI-II was used [97]. Our sample yielded a McDonald’s omega value of 0.84 for the cyber-victimization subscale.

#### The CAPE-42

In this study, we used the positive dimension of the CAPE, which contains 20 out of 42 total items of the scale [98]. The positive CAPE dimension assesses positive SRPEs on a two-dimensional scale: (1) frequency of SRPEs and (2) degree of distress caused by them. This 20-item positive dimension can be divided into four types of positive SRPEs: Bizarre Experiences, Perceptual Abnormalities, Persecutory Ideation, and Magical Thinking. We only used the total scores of the frequency sub-dimension of the positive CAPE dimension, with scores ranging from 20 to 80. The Arabic version of scale yielded excellent psychometric properties [99], with McDonald’s omega value being calculated as 0.78 for the positive CAPE dimension subscale used in this study.

#### The DASS-8

The DASS-8 [100] is an Arabic version of the DASS, composed of eight items and three dimensions: anxiety (e.g., “felt scared without reason”; three items), depression (e.g., “felt down hearted and blue”; three items), and stress (e.g., “was using a lot of my mental energy”; two items). In the present sample, McDonald’s omega values were the following: depression (0.73), anxiety (0.73) and stress (0.65).

#### The ISI

This a reliable measure for evaluating the nature, severity and impact of insomnia symptoms [70]. It is composed of 7 self-report items assessing the following sleep parameters: sleep maintenance and early morning awakening problems, severity of sleep onset, noticeability of sleep problems by others, interference of sleep difficulties with daytime functioning, sleep dissatisfaction, and distress caused by the sleep difficulties. The Arabic version of the ISI has been used [72] (McDonald’s omega in this study = 0.82).

### Statistical analysis

SPSS software version 23 was used to conduct data analysis. We had no missing data in our database. McDonald’s omega values were recorded for reliability analysis of all scales and subscales. The positive dimension score was normally distributed as its skewness and kurtosis values varied between − 1 and + 1 [101]; therefore, the Student t test was used to compare two means, ANOVA test to compare three or more means, and the Pearson test to correlate two continuous variables. To check for a significant indirect effect of insomnia severity/psychological distress between cyber-victimization and positive dimension, we conducted a mediation analysis using SPSS PROCESS v3.4 model 4 with three pathways; pathway A from the independent variable to the mediator, pathway B from the mediator to the dependent variable and pathway C from the independent to the dependent variable. Variables that showed a p < 0.25 in the bivariate analysis were entered in the path analysis. Significance was set at a p < 0.05.

## Results

A total of 4158 Lebanese and 735 Tunisian participants filled the survey; 1055 Lebanese and 72 Tunisians were excluded for having self-reported mental physician-diagnosed mental health issues. Finally, the total sample was composed of 3766 participants; 3103 were from Lebanon and 663 from Tunisia. A higher mean age was significantly found in the Tunisian sample, whereas higher insomnia severity and psychological distress mean scores were significantly found in the Lebanese sample. No significant difference was found in terms of gender between the two groups. All sociodemographic and other characteristics of the participants are summarized in Table 1.

**Table 1:** *Sociodemographic and other characteristics of the participants (N = 3103)*

Variable	Lebanon (n = 3103)	Tunisia (n = 663)	*p*
Gender			0.073
Male	1130 (36.4%)	266 (40.1%)	
Female	1973 (63.6%)	397 (59.9%)	
Marital status			**< 0.001**
Single	2800 (90.2%)	551 (83.1%)	
Married	303 (9.8%)	112 (16.9%)	
Education			**< 0.001**
Secondary or less	159 (5.1%)	438 (66.1%)	
University	2944 (94.9%)	225 (33.9%)	
Housing area			**< 0.001**
Urban	1498 (48.3%)	622 (93.8%)	
Rural	1605 (51.7%)	41 (6.2%)	
Living arrangement			**< 0.001**
Alone	117 (3.8%)	537 (81.0%)	
With family	2962 (95.5%)	112 (16.9%)	
With friends	24 (0.8%)	14 (2.1%)	
Cigarettes smoking			**< 0.001**
No	2749 (88.6%)	323 (48.7%)	
Yes	354 (11.4%)	340 (51.3%)	
Alcohol drinking			**< 0.001**
No	2645 (85.2%)	466 (70.3%)	
Yes	458 (14.8%)	197 (29.7%)	
Cannabis use			**< 0.001**
No	3066 (98.8%)	516 (77.8%)	
Yes	37 (1.2%)	147 (22.2%)	
Other illegal drug use			**< 0.001**
No	3083 (99.4%)	545 (82.2%)	
Yes	20 (0.6%)	118 (17.8%)	
	**Mean ± SD**	**Mean ± SD**	
Age (in years)	21.73 ± 3.80	26.32 ± 4.86	**< 0.001**
Household crowding index (person/room)	1.51 ± 0.72	2.07 ± 1.16	**< 0.001**
Insomnia severity index	9.07 ± 6.04	7.77 ± 2.73	**< 0.001**
Psychological distress	6.21 ± 5.50	5.50 ± 1.68	**< 0.001**
Cyber-victimization	12.71 ± 3.92	5.99 ± 2.90	**< 0.001**

Note. CAPE: Community Assessment of Psychic Experiences

### Bivariate analysis

The results of the bivariate analysis are summarized in Tables 2 and 3. Higher positive dimension scores were found in Lebanese participants compared to Tunisians, in single participants compared to married ones, in those with a university level of education compared to secondary or less, in those who live in rural areas compared to urban, in those who do not smoke, do not drink alcohol and do not use marijuana or any other illegal drug. Furthermore, more cyber-victimization, a higher insomnia severity and psychological distress were significantly associated with higher positive dimension scores. Finally, older age was significantly associated with lower positive dimension scores.

**Table 2:** *Bivariate analysis of factors associated with the CAPE positive dimension*

Variable	Positive psychotic experiences	*P*	t	*df*
Country		**< 0.001**	19.14	3764
Lebanon	31.59 ± 6.27			
Tunisia	26.50 ± 5.95			
Gender		0.201	1.30	3764
Male	30.51 ± 6.74			
Female	30.80 ± 6.38			
Marital status		**0.028**	2.19	3764
Single	30.77 ± 6.52			
Married	30.03 ± 6.44			
Education		**< 0.001**	10.41	3764
Secondary or less	28.18 ± 6.66			
University	31.17 ± 6.38			
Housing area		**< 0.001**	7.28	3764
Urban	30.02 ± 6.69			
Rural	31.56 ± 6.17			
Living situation		**< 0.001**	105.76	3765
Alone	27.51 ± 6.38			
With family	31.41 ± 6.34			
With friends	27.74 ± 5.35			
Cigarettes smoking		**< 0.001**	5.11	3764
No	30.95 ± 6.29			
Yes	29.55 ± 7.31			
Alcohol drinking		**< 0.001**	5.17	3764
No	30.94 ± 6.43			
Yes	29.50 ± 6.79			
Marijuana use		**< 0.001**	10.41	3764
No	30.94 ± 6.38			
Yes	25.89 ± 7.16			
Other illegal drug use		**< 0.001**	11.49	3764
No	30.93 ± 6.45			
Yes	24.54 ± 4.83			

Numbers in bold indicate significant *p* values

**Table 3:** *Correlation of continuous variables with the CAPE positive dimension*

	1	2	3	4	5	6
1. Positive dimension	1					
2. Age	− 0.09***	1				
3. Household crowding index	− 0.01	− 0.01	1			
4. Cyber-victimization	0.44***	− 0.23***	− 0.16***	1		
5. Insomnia severity index	0.37***	− 0.01	0.001	0.24***	1	
6. Psychological distress	0.41***	− 0.004	0.01	0.29***	0.50***	1

***p < 0.001

### Mediation analysis

**Fig. 1:**
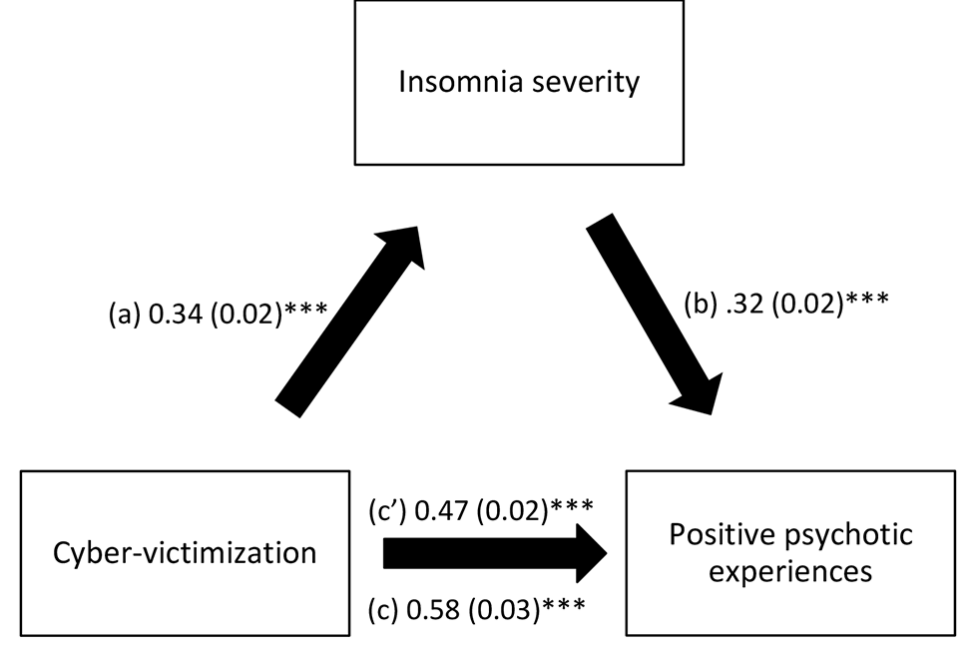
(**a**) Relation between cyber-victimization and insomnia severity (R2 = .068); (**b**) Relation between insomnia severity and CAPE positive dimension (R2 = .283); (**c**) Total effect of cyber-victimization on positive psychotic experiences (R2 = .211); (c’) Direct effect of cyber-victimization on positive psychotic experiences. Numbers are displayed as regression coefficients (standard error). ***p < 0.001

**Fig. 2:**
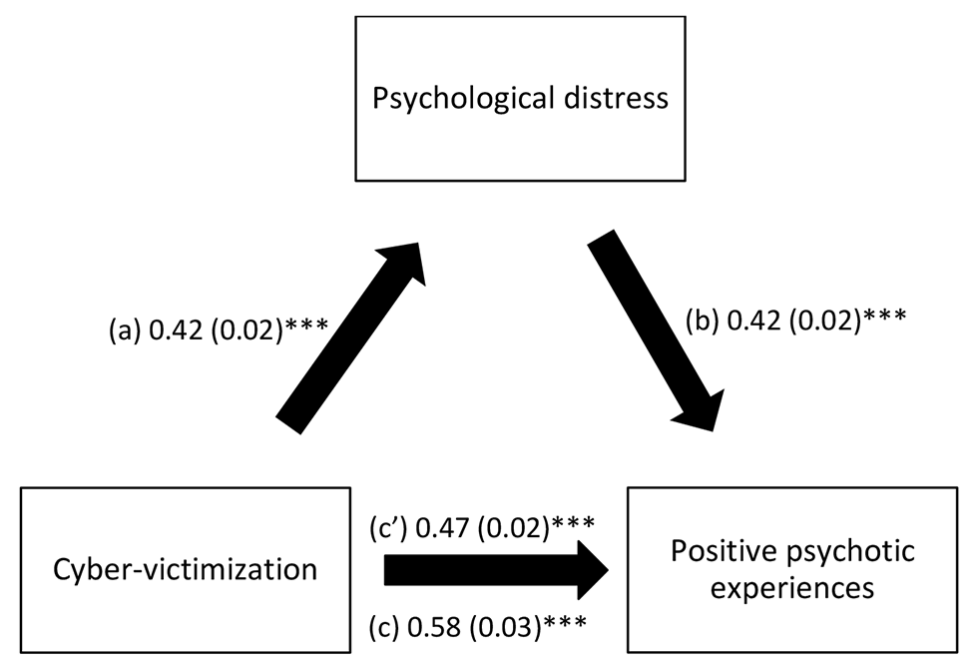
(**a**) Relation between cyber-victimization and psychological distress (R2 = .118); (**b**) Relation between psychological distress and CAPE positive dimension (R2 = .304); (**c**) Total effect of cyber-victimization on positive psychotic experiences (R2 = .211); (c’) Direct effect of cyber-victimization on positive psychotic experiences. Numbers are displayed as regression coefficients (standard error). ***p < 0.001

The results of the mediation analysis showed that insomnia severity and psychological distress mediated the association between cyber-victimization and positive dimension (Table 4). Higher cyber-victimization was significantly associated with more insomnia severity/psychological distress, which were, in turn, significantly associated with more positive dimension. Finally, more cyber-victimization was significantly and directly associated with more positive dimension (Figs. 1 and 2).

**Table 4:** *Mediation analyses results, taking the cyber-victimization score as the independent variable, insomnia severity/psychological distress as the mediator and positive dimension score as the dependent variable*

	Direct effect			Indirect effect		
	Beta	SE	*P*	Beta	Boot SE	Boot CI
Insomnia severity	0.47	0.02	< 0.001	0.11	0.01	0.09; 0.13*
Psychological distress	0.40	0.03	< 0.001	0.18	0.01	0.15; 0.20*

*indicates significant mediation; results were adjusted over the following variables: country, gender, marital status, education level, living situation, alcohol drinking, cannabis use, housing area, cigarette smoking, other illegal drug use, and the other two mediators

## Discussion

We build on the extent literature on face-to-face victimization to theorize about the nature and the mediating factors in the relationship between cybervictimization and psychosis proneness. As such, we sought to examine the hypothesized indirect effects of cybervictimization on SRPEs through insomnia and psychological distress. Findings indicate that our hypothesis is partly supported. After controlling for potential confounders (including country of origin, sociodemographic variables and substance use), analyses revealed that insomnia and distress symptoms were partial mediators of the relationship cybervictimization-SRPEs.

As for the direct effects, our results demonstrate that cybervictimization significantly and positively correlates with SRPEs in non-clinical individuals from the MENA region. These findings are in agreement with the limited existing literature. A study published in 2022 by Paruk et al. [45] surveyed South African adolescents aged 13–18 years, and showed that schizophrenia represented the second most frequent psychiatric diagnosis associated with cyber-victimization (57.1%), following major depressive disorder (72.4%). In another study by Magaud et al. [46] revealed that cyber-victimization, as assessed using self-developed questions, was highly prevalent (38%) among individuals at clinical high risk (CHR) for psychosis, and mostly received via text messages, instant messaging and Facebook. A recent Chinese study among high school students reported that involvement in cyberbullying either as victims or as bullies was significantly linked to SRPEs [47]; whereas a Turkish study among undergraduate students found that engaging in cyberbullying as a perpetrator was associated with greater psychoticism [48]. Our findings preliminarily confirm that the well-established relationship between victimization and psychosis would apply to the cyber form of victimization; and that this relationship also applies to other previously unexplored cultural backgrounds. However, given that our data is cross-sectional and that this is the first study to investigate cybervictimization in relation to psychotic symptoms in an Arab country from the MENA region, the present results should be considered tentative pending future longitudinal studies in the same context.

Regarding the indirect effects, we found, as expected, that the two theoretically-driven factors, i.e. psychological distress symptoms and insomnia severity, partially mediated the association between cybervictimization and SRPEs. This important finding is consistent with the fact that both psychological distress and sleep problems have been suggested as potential consequences of cyber-bullying [62, 63, 66, 73, 75]; and both have been hypothesized as precipitating and perpetuating risk factors for psychotic symptoms [102–105]. A mediator refers to an intermediate variable “which represents the generative mechanisms through which the focal independent variable is able to influence the dependent variable of interest” [106]. Mediators thus enable a comprehensive understanding of the mechanism linking cybervictimization to SRPEs. Findings cautiously suggest that untreated insomnia and distress symptoms might add additional vulnerability to cyber-victims, contributing thereafter to more severe positive psychotic symptoms. It is of note, however, that the relationship between cyberbullying, insomnia and SRPEs seem to be complex and multi-determined. Research found that sleep deprivation leads to decreased activity in brain areas of the theory of mind neural network and increased activity in areas involved in perceiving threatening approach, which, in turn, results in social withdrawal [107]. Sleep insufficiency has also been demonstrated to increase amygdala activation [108–110], which is involved in threat perception (e.g., [111, 112]). We thus suggest that insomnia may partly contribute to misinterpreting neutral or ambiguous stimuli in the cyber-environment as hostile and threatening. Interestingly, abnormal response to neutral stimuli during emotional processing tasks and increased activity in temporal cortex have also been reported in at -risk for psychosis populations [113]. This suggests that an individual with psychosis may experience neutral or benign cyber-communication as negatively directed toward them. These observations highlight that future research is needed to investigate neural mechanisms that may underscore the connection between cyberbullying, insomnia and SRPEs. More longitudinal studies are also required to elucidate the causal relationships between these variables. The future studies need to go beyond self-report measures and take into account behavioral/clinical aspects of cyberbullying, as well as objective measures of insomnia.

### Limitations

Before drawing any conclusions from our findings, certain limitations need to be discussed. First, our data is cross-sectional; which implies that any estimations of mediation effects are correlational in nature, and the correct causal ordering assumption cannot be tested until longitudinal research is conducted. Second, the generalizability of findings may be limited, because our sample was based on a web-based convenience sampling. Third, our results may be subject to response biases due to self-report method effects. This may be a concern, especially since symptoms of insomnia, depression and anxiety may overlap (e.g., [114, 115]). To address this limitation, future studies need to consider using objective measures of sleep disturbances and structured clinical interviews to assess depression and anxiety levels. A fourth limitation lies to the fact that, expect for gender, all other sociodemographic variables (i.e., age, marital status, education level, housing area, living arrangement, substances use) were statistically significant between the Tunisian and Lebanese samples. These heterogeneous characteristics of the two samples might have affected our findings. We highlight, however, that all these factors were controlled for in the mediation analyses. Finally, further studies should consider investigating the role of other mediators on the interplay between cybervictimization and positive SRPEs, such as alexithymia and difficulties in emotional regulation [116–118].

### Study implications

The current findings that cybervictimization is positively correlated with SRPEs add further support to the association between victimization, in general, and psychosis proneness. In addition, findings shed light on the significant indirect role of insomnia and distress in the cross-sectional link between cybervictimization and positive SRPEs. This preliminarily suggests that these psychopathological factors may be regarded as potential prevention and early-intervention targets for psychosis. Therefore, we recommend, with the caution appropriate to the cross-sectional design, that screening and monitoring for insomnia, depression, anxiety, and stress be incorporated into the routine mental health examination for individuals exposed to cybervictimization who present with SRPEs; and when appropriate, interventions should be delivered. Sufficient evidence has been adduced to confirm the effectiveness of sleep interventions, such as proper sleep hygiene and tracking [119], or Cognitive Behavioral Therapy for insomnia (CBTi) [120–122] in individuals with early and subthreshold psychosis (for review, see [104]). Interestingly, the CBTi has proven beneficial in improving attenuated psychotic symptoms [120, 123]. In this line of thinking, it has also been observed that antidepressants may have an antipsychotic action through improvement of mood state and reduction of inadequate appraisal of attenuated positive symptoms [124]. Fusar-Poli et al. [124] proposed that antidepressants could affect individuals’ psychosis risk by modulating their response to environmental stresses, either directly through neurochemicals implicated in controlling responses to stress, or indirectly by preventing anxiety/depression subsequent to these stresses. The above-mentioned interventions can act as buffers to prevent psychosis in cyber-victimized young people with pre-existing genetic predisposition.

## Conclusion

The current research had denoted for the first time that insomnia and psychological distress have a possible mediating role in the cybervictimization/positive SRPEs connection. Identifying these mediators could provide novel insight for psychosis prevention efforts and intervention targets for cyber-victimized individuals prone to experience subclinical psychotic symptoms. Our findings also offer an empirical basis for future longitudinal research on the nature and mechanisms of the relationship between cyber forms of victimization and psychosis in healthy young individuals.

## Data Availability

All data generated or analyzed during this study are not publicly available due the restrictions from the ethics committee. Reasonable requests can be addressed to the corresponding author.
